# Natural Endogenous Human Matriptase and Prostasin Undergo Zymogen Activation via Independent Mechanisms in an Uncoupled Manner

**DOI:** 10.1371/journal.pone.0167894

**Published:** 2016-12-09

**Authors:** Hui Chen Su, Yan A. Liang, Ying-Jung J. Lai, Yi-Lin Chiu, Robert B. Barndt, Frank Shiao, Hsiang-Hua D. Chang, Dajun D. Lu, Nanxi Huang, Chun-Che Tseng, Jehng-Kang Wang, Ming-Shyue Lee, Michael D. Johnson, Shih-Ming Huang, Chen-Yong Lin

**Affiliations:** 1 Department of Pharmacy, Chi-Mei Medical Center, Tainan, Taiwan; 2 Lombardi Comprehensive Cancer Center, Department of Oncology Georgetown University, Washington DC, United States of America; 3 Department of Biochemistry, National Defense Medical Center, Taipei, Taiwan; 4 Graduate Institute of Biochemistry and Molecular Biology, College of Medicine National Taiwan University, Taipei, Taiwan; Florida State University, UNITED STATES

## Abstract

The membrane-associated serine proteases matriptase and prostasin are believed to function in close partnership. Their zymogen activation has been reported to be tightly coupled, either as a matriptase-initiated proteolytic cascade or through a mutually dependent mechanism involving the formation of a reciprocal zymogen activation complex. Here we show that this putative relationship may not apply in the context of human matriptase and prostasin. First, the tightly coupled proteolytic cascade between matriptase and prostasin might not occur when modest matriptase activation is induced by sphingosine 1-phospahte in human mammary epithelial cells. Second, prostasin is not required and/or involved in matriptase autoactivation because matriptase can undergo zymogen activation in cells that do not endogenously express prostasin. Third, matriptase is not required for and/or involved in prostasin activation, since activated prostasin can be detected in cells expressing no endogenous matriptase. Finally, matriptase and prostasin both undergo zymogen activation through an apparently un-coupled mechanism in cells endogenously expressing both proteases, such as in Caco-2 cells. In these human enterocytes, matriptase is detected primarily in the zymogen form and prostasin predominantly as the activated form, either in complexes with protease inhibitors or as the free active form. The negligible levels of prostasin zymogen with high levels of matriptase zymogen suggests that the reciprocal zymogen activation complex is likely not the mechanism for matriptase zymogen activation. Furthermore, high level prostasin activation still occurs in Caco-2 variants with reduced or absent matriptase expression, indicating that matriptase is not required and/or involved in prostasin zymogen activation. Collectively, these data suggest that any functional relationship between natural endogenous human matriptase and prostasin does not occur at the level of zymogen activation.

## Introduction

The type 2 transmembrane serine protease matriptase and the glycosylphosphatidylinositol (GPI)-anchored serine protease prostasin contribute to the formation of the epidermal barrier in mouse skin apparently by working together in a tightly coupled proteolytic cascade. Support for this conclusion comes from several lines of evidence: 1) Targeted deletion of matriptase or prostasin results in almost identical epidermal defects in the skin of the respective knockout mice [1,2]. 2) Matriptase and prostasin are co-expressed in the uppermost layer of viable keratinocytes in the mouse epidermis and so could act in concert to contribute to the later stages of epidermal differentiation and the formation of the epidermal barrier [3]. 3) Prostasin zymogen activation does not appear to occur in the skin of matriptase knockout mice [3]. These data, along with the observation that activation of human matriptase zymogen occurs by autoactivation [4], support the conclusion that matriptase and prostasin function as a proteolytic cascade with matriptase as an upstream activator and prostasin as a downstream substrate contributing to epidermal barrier formation. Both matriptase and prostasin are synthesized as zymogen forms [5–7]. While matriptase zymogen exhibits unusually high intrinsic activity and could play an important role in matriptase autoactivation [8] neither matriptase or prostasin zymogens form stable complexes with their cognate inhibitors, hepatocyte growth factor activator inhibitor (HAI)-1 and HAI-2 [9,10]. After activation, however, both proteases acquire proteolytic activity and the ability to form very high affinity complexes with the HAI proteins. Further evidence for the matriptase-prostasin cascade is, therefore, provided using HaCaT human keratinocytes. In these cells, matriptase and prostasin zymogen activation occurs in lockstep with the simultaneous activation of the proteases being immediately followed by rapid HAI-1-mediated inhibition of both active matriptase and active prostasin [9]. Thus, the apparently tightly coupled proteolytic cascade is believed to be initiated by autoactivation of the matriptase zymogen to generate active enzyme, which subsequently activates prostasin [3]. The active prostasin produced could then act as the sole downstream effector of matriptase, participating directly in the formation of the epidermal barrier of mouse skin.

This model of a one-way cascade relationship between the proteases with matriptase activating prostasin has, however, been challenged by several studies that suggest that there is a much more complicated relationship regarding their zymogen activation. In mouse placenta, prostasin appears to be required for matriptase zymogen activation and prostasin activation is not matriptase-dependent [11]. In solution, recombinant active prostasin at concentrations as high as 500 nM fails to activate matriptase variants with a non-functional serine protease domain [12]. In contrast, when recombinant active prostasin is added exogenously to Caco-2 cells or HaCaT human keratinocytes, it can activate matriptase at much lower concentrations (5 or 50 nM respectively) than those that failed to activate matriptase in solution (500 nM) [12,13]. Exogenously induced prostasin co-expression in HEK293 cells increases matriptase zymogen activation [12], whereas high levels of prostasin expressed significantly reduces matriptase expression [14]. Thus, data from reconstituted cell-based systems in which prostasin is added in some way to cells suggest that prostasin can function as an upstream matriptase activator and/or inducer of matriptase zymogen activation, even though prostasin does not activate matriptase efficiently in solution. An interesting hypothesis has been proposed to reconcile these data and the previous one-way proteolytic cascade model. This suggests that prostasin may act as a co-factor for matriptase zymogen activation through the formation of a reciprocal zymogen activation complex between matriptase and prostasin [14]. Furthermore, it has been suggested that prostasin proteolytic activity is not required for its role as the cofactor in the matriptase zymogen activation complex.

The complexity of the relationship between matriptase and prostasin illustrated by this confusing literature may result in part from the matriptase autoactivation mechanism which can be induced in different contexts by a variety of factors, including steroid hormones, lysophospholipids, chemicals, protein factor, and cellular chemical environments, such as low pH and oxidative stress [15–22]. The mechanisms regulating prostasin zymogen activation remain largely to be explored with the exception of the potential role of matriptase as the upstream activator. The abovementioned studies examining the functional relationship between matriptase and prostasin have involved genetic manipulation of cell systems or animals and some of the confusion in the literature may be the result of non-physiological protein expression levels or altered sub-cellular localization, and / or activation of the proteins in these manipulated models. Given the important physiological roles played by both matriptase and prostasin, defining and/or clarifying the functional relationship between these two serine proteases will provide insights into how the membrane-associated proteolysis is regulated and contribute to physiological processes. In the current study, the functional relationship between these enzymes is explored at the zymogen activation levels by focusing on the ability of matriptase and prostasin to undergo spontaneous and induced zymogen activation in a variety of human cells that endogenously express both proteases, or matriptase or prostasin alone. Our data show that matriptase can undergo zymogen activation in the absence of prostasin, and *vice versa*, and that significant spontaneous prostasin zymogen activation can occur without matriptase involvement. It is very likely that matriptase and prostasin can undergo zymogen activation through independent and unrelated mechanisms in the majority of human cells.

## Materials and Methods

### Chemicals and reagents

5,5’-Dithio-bis-(2-Nitrobenzoic Acid) (DTNB) was obtained from Sigma-Aldrich (St. Louis, MO); Fetal bovine serum (FBS) was obtained from Omega Scientific (Tarzana, CA).

#### Cell cultures

The human prostate epithelial cell line RWPE-1 (ATCC) was cultured in keratinocyte serum free medium (Invitrogen) supplemented with 50 μg/ml bovine pituitary extract and 5 ng/ml recombinant human epidermal growth factor (rhEGF). The human mammary epithelial cell line184 A1N4 (a gift from M. R. Stampfer, UC Berkeley) [23] was cultured in modified Improved Minimum Essential Medium (IMEM) supplemented with 0.5% FBS, 5 μg/ml recombinant human insulin (rh-insulin) (Invitrogen), 5 μg/ml hydrocortisone (Sigma), and 10 ng/ml rhEGF (Promega). The milk-derived human mammary epithelial cell lines MTSV-1.1 B and MTSV-1.7 (a gift from Dr. J. Taylor-Papadimitriou, Imperial Cancer Research Fund, London) [24] were cultured in IMEM supplemented with 10% FBS. The human prostate cancer cell lines DU145 and PC3 and the human hematological cancer cell lines Raji, Namalwa, and Ramos (all from ATCC) were cultured in RPMI-1640 medium supplemented with 10% FBS. Caco-2 human enterocytes, Heb3B human hepatoma, and ACHN human renal adenocarcinoma cell lines (all from ATCC) were cultured in DMEM supplemented with 10% FBS. All cells were incubated at 37°C in a humidified atmosphere with 5% CO_2_.

### Monoclonal antibodies

M24 anti-human matriptase monoclonal antibody (mAb) was used for immunoblot analyses to detect total matriptase, the use and validation of which has been previously reported [20,25,26]. The mouse mAb M19 was used for immunoblot analyses to detect HAI-1, as we have previously described [20,25,26]. The mouse mAbs YL10 and YL11 were used to detect human prostasin as previously described [10]. The detection sensitivity is estimated to be around 0.1–0.2 ng for matriptase mAb [27], 0.5–1 ng for HAI-1 mAb [25], and 0.1–0.2 ng for prostasin mAbs.

### Induction of matriptase zymogen activation

For the induction of matriptase zymogen activation, cells grown in 100 mm dishes were washed with PBS three times and then incubated at room temperature with 150 mM phosphate buffer pH 6.0 or with phosphate buffered saline (PBS) (non-activation control). After 20 min, the cells were washed with PBS, scraped from the dishes in PBS, and the cells pelleted by centrifugation at 10,000 rpm using an Eppendorf tabletop centrifuge for 1 min. The cell pellets were lysed in 1% Triton X-100 and 1 mM DTNB in PBS. DTNB was added to the lysis buffer to prevent cleavage of disulfide linkages [17].

### Western blotting

The protein concentration of the cell lysates were determined by Bradford protein assay and equal amounts of protein of cell lysates were analyzed by Western blot as indicated below. Protein samples were diluted in 5x sample buffer containing no reducing agent and incubated at room temperature for 5 min. Proteins were resolved by 7.5% SDS-PAGE, transferred to nitrocellulose membranes, and probed with the indicated mAbs. The binding of mAbs was detected using HRP conjugated secondary antibodies, and visualized using the Western Lightening^®^ Chemiluminescence Reagent Plus (Perkin-Elmer, Boston, MA).

### CRISPR design

The first exon of SPINT2 (nucleotide 292 to 541 of NM_001166103) was queried using the CRISPR Target Finder algorithm (http://crispr.mit.edu/) to identify two guide RNAs (gRNA) with unique nickase sites. In order to generate constructs for each gRNA in pX335 (Addgene), the following oligonucleotides (IDT) were designed and annealed as previously described [28]:

gRNA1 antisense 5’AAACGATCGCGAGACCCCAACGGC3’

gRNA1 sense 5’CACCGCCGTTGGGGTCTCGCGATC3’

gRNA2 antisense 5’AAACTGGCCAGCTCAGCCGAGACGC3’

gRNA2 sense 5’CACCGCGTCTCGGCTGAGCTGGCCA3’

Plasmid DNA for each gRNA construct was prepared using the HiSpeed Plasmid Midi kit following the manufacturer’s instructions (Qiagen), and sequenced prior to transfection.

#### Transfection and selection

Caco-2 cells were plated at 30–40% confluency on 10 cm dishes (Corning) 48 hours prior to transfection. Cells were then co-transfected with each gRNA construct and pEF6/V5 His (Invitrogen) using Lipofectamine 2000 according to the manufacturer’s instructions (LifeTechnologies) and as previously described (http://www.bio-protocol.org/e130). Briefly, 1.125 pmol of total plasmid DNA in a 0.5:0.5:0.2 ratio (gRNA1sense:gRNA1antisense:pEF6/V5 His) was added to 500 μl OptiMEM (LifeTechnologies). Separately, 40 μl of Lipofectamine 2000 was added to 500 μl OptiMEM. These two solutions were combined and incubated at room temperature for 45 minutes. The media on the cells was replaced with 2.5 ml of serum-free DMEM, and the transfection mixture volume was raised to 2.5 ml with OptiMEM and then added to the cells dropwise. Six hours later 5 ml of DMEM supplemented with 20% FBS was added to the cells. The cells were grown for another 48 hours and then split into 10 cm dishes at 1:1, 1:10, 1:100 dilutions in the presence of 10 μg/mL Blasticidin (LifeTechnologies) and cultured for 3–4 weeks. Individual clones were isolated by gently scraping the cells from the dish and immediate drawing them up with a blunted and beveled pipette tip. The cells in <10 ul PBS were then placed into a well of a flat-bottomed 96-well plate (Corning) with 30 ul 0.05% Trypsin-0.53 mM EDTA (Sigma) for 5–10 minutes in a 37°C incubator. The cells were dispersed by gentle pipetting and transferred to a new 96-well plate with 200 ul complete media without Blasticidin. Clones were expanded over the next 3–6 weeks.

### Screening for HAI-2 expression

Cells were washed with PBS then lysed with RIPA buffer (10 mM Tris-HCl (pH 8.0), 1 mM EDTA, 0.5 mM EGTA, 1% Triton X-100, 0.1% sodium deoxycholate, 0.1% SDS, 140 mM NaCl) directly on the dish. Lysates were centrifuged for 10 minutes at 4°C, and the supernatant was retained, and the protein concentration determined using the Pierce BCA Protein Assay (ThermoScientific) and read on an ELx808 microplate reader (BioTek). Equal amounts of lysate (non-boiled, non-reducing) diluted with 5X sample loading buffer were fractionated by 10% SDS-PAGE, transferred to Amersham/Protran 0.45 um nitrocellulose (GE Healthcare), and then blocked with 5% non-fat milk in PBS/0.1%Tween20 (Sigma). Blots were probed with 1:2000 dilution of the anti-HAI-2 monoclonal DC16 [29,30] and imaged as described above.

### Analysis of CRISPR-generated alleles

Genomic DNA was isolated from individual clones. Cells were harvested by trypsinization, washed with PBS, centrifuged at 5000 rpm for 5’ at room temperature in a microcentrifuge, and the supernatant discarded to yield a pellet of approximately 0.5 million cells. The cells were resuspended in 200 ul of digestion buffer (50 mM Tris-HCl (pH 8.0), 10 mM EDTA, 50 mM NaCl, 0.5% SDS, 0.5 μg/ul proteinase K (LifeTechnologies)) and incubated for 2 hr at 37C. An equal volume of digestion buffer without proteinase K was added, and the lysates were extracted twice with an equal volume of 1:1 phenol:chloroform (Sigma), and once with chloroform. DNA was precipitated by the addition of two volumes of 100% ethanol, collected by centrifugation and the pellet washed with 70% ethanol, before air-drying and resuspension in water. The DNA concentration was determined and 100 ng of genomic DNA was used as a PCR template with the following oligos (IDT):

5’ GCG AGT GAG GAG CAG ACC 3’

5’ GCT CCC AAA CCT CAT TTC AA 3’.

OneTaq 2X Master Mix (NEB) was used according to the manufacturer’s instructions and with the following program: 95C 2’ 1 cycle; 95C 30s, 54C 30s, 72C 1’ for 35 cycles; 72C 5’; 4C 15’, on an MJ mini personal thermocycler (BioRad). PCR products were isolated from 1.5% LMP/ 1X TBE agarose gel using the QIAquick gel extraction kit (Qiagen) and sequenced using the same PCR primers.

## Results

### Induction of robust matriptase zymogen activation leads to simultaneous prostasin zymogen activation in human mammary and prostate epithelial cells

**Fig 1:**
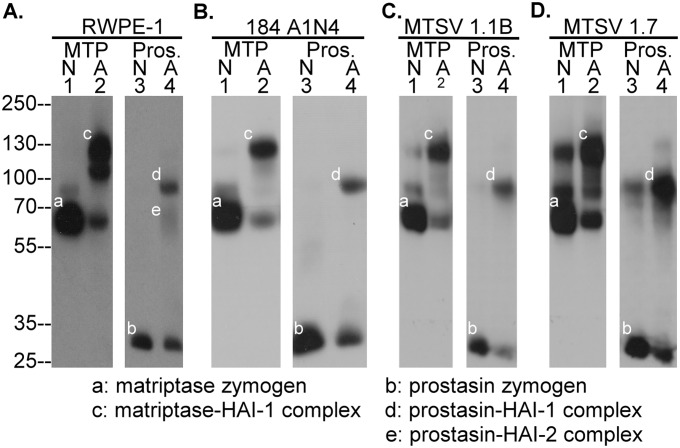
Human mammary and prostate epithelial cells simultaneously activate matriptase and prostasin in response to transient exposure to a pH 6.0 buffer. Human prostate epithelial cells RWPE-1 (*A*.), mammary epithelial cells 184 A1N4 (*B*.), MTSV 1.1B (*C*.) and MTSV 1.7 (*D*.) were exposed to PBS as a non-activation control (N, lanes 1 and 3) or phosphate buffer pH 6.0 for 20 min (A, lanes 2 and 4). Cell lysates were prepared and analyzed by Western blot for species containing matriptase (MTP, lanes 1 and 2, bands *a* and *c*) or prostasin (Pros., lanes 3 and 4, bands *b*, *d*, and *e*). This experiment was conducted at least twice with the same profile observed. Representative data are shown.

Induction of simultaneous matriptase and prostasin zymogen activation, followed by rapid HAI-1-mediated inhibition of the nascent active matriptase and prostasin, has been observed in HaCaT human keratinocytes when the cells were transiently exposed to a pH 6.0 buffer [9]. The same phenomenon can be induced in human prostate epithelial cells RWPE-1(Fig 1A), human mammary epithelial cells 184 A1N4 (Fig 1B), MTSV1.1B (Fig 1C), and MSTV1.7 (Fig 1D). All of these cell lines express both matriptase and prostasin, primarily in their zymogen forms: 70-kDa for matriptase (Fig 1, lanes 1, band *a*) and 32-kDa for prostasin (Fig 1, lanes 3, band *b*), in lysates prepared from these cells when maintained in their usual respective culture media. Following exposure of the cells to a pH 6.0 buffer for 20 min, significant amounts of both the matriptase and prostasin zymogen are converted to their activated forms of the enzymes which can be detected in lysates prepared from all of the lines as complexes with HAI-1 (Fig 1, lanes 2 and 4, bands *c* and *d*, respectively). Activated prostasin in complex with HAI-2 was also observed in RWPE-1 cell lysates as a diffuse band with a size smaller than the 70-kDa molecular weight marker (Fig 1A, lane 4, *e*). These data suggest that simultaneous matriptase and prostasin zymogen activation can be induced not only in stratified epithelial cells, such as keratinocytes, but also in simple epithelia cells, such as mammary and prostate epithelial cells.

### Matriptase zymogen activation is not necessary followed by significant prostasin zymogen activation

**Fig 2:**
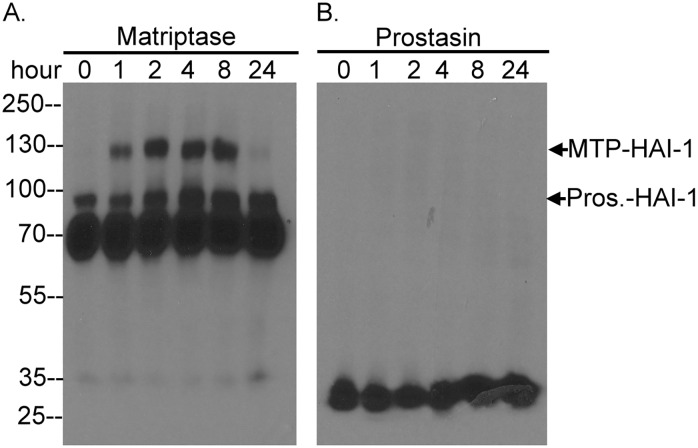
Sphingosine 1-phosphate induces matriptase activation without inducing prostasin zymogen activation. Human mammary epithelial cells 184 A1N4 were treated with fresh medium, which contains sphingosine 1-phosphate derived from FBS, for the indicated times. The cells were harvested and analyzed by Western blot for matriptase species (*A*) and prostasin (*B*.). The size of matriptase-HAI-1 complex (MTP-HAI-1) and prostasin-HAI-1 complex (Pros.-HAI-1) were indicated. The experiment was conducted twice with the same profile observed. Representative data are shown.

Acid-induced matriptase zymogen activation is a biochemical event which has been shown to occur in cell homogenates in which the continued association of matriptase with the insoluble membrane fraction of the cell homogenate is sufficient for the induction of zymogen activation [19]. As noted above, this phenomenon can be observed using live cells which exhibit robust matriptase zymogen activation in response to exposure to a mildly acidic environment [20]. Matriptase zymogen activation is also impacted by other cellular chemical factors such as chloride ions; physiologically relevant concentration of which can significantly attenuate acid-induced matriptase zymogen activation [21]. For this reason matriptase zymogen activation may not occur *in vivo* to the levels observed in the acid-induced model as described in Fig 1, and in fact matriptase activation appears to be maintained at a very low levels in most normal organ systems with exception of some stomach cells [31]. We, therefore, next set out to investigate whether prostasin zymogen activation still occurs when matriptase zymogen activation is induced under much more physiological conditions by the lysophospholipid sphingosine 1-phosphate (S1P) in 184 A1N4 human mammary epithelial cells [15,16]. As shown in Fig 2A, matriptase zymogen activation was induced to a more modest extent one hour after exposing the cells to fresh culture medium and activated matriptase in complex with HAI-1 remained associated with the cells for at least 8 hours post exposure. In contrast, in spite of the matriptase activation, there was no evidence of prostasin activation due to the absence of detectable activated prostasin complex with HAI-1 being observed regardless of the status of matriptase zymogen activation (Fig 2B). The lack of obvious prostasin zymogen activation when more modest levels of matriptase zymogen activation were induced suggests that the extent of matriptase zymogen activation may be an important determinant in the simultaneous of prostasin zymogen activation. This observation suggests that while prostasin may be a downstream substrate of matriptase, prostasin zymogen activation is not necessary closely coupled with matriptase autoactivation.

### Prostasin does not participate in acid-induced matriptase autoactivation

**Fig 3:**
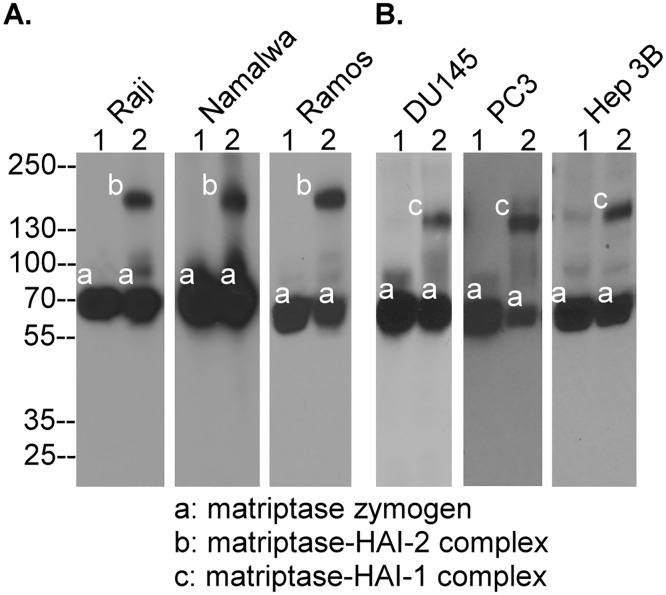
Matriptase can undergo autoactivation in cells that do not express prostasin. Human hematological cancer cell lines Raji, Namalwa, and Ramos, human prostate cancer cell lines DU145 and PC3, and human hepatoma Hep3B cells were treated with PBS as the non-activation control (lanes 1) or pH 6.0 buffer for 20 min to induce matriptase activation. Cell lysates were prepared and analyzed by Western blot analysis for matriptase species using the matriptase specific mAb M24. The matriptase species observed include matriptase zymogen (band *a*), matriptase-HAI-2 complex (band *b*), and matriptase-HAI-1 complex (band *c*). This study was conducted multiple times (n>5) with the same profile observed. Representative data are shown.

It has been proposed that prostasin participates in matriptase autoactivation through the formation of a reciprocal zymogen activation complex [14]. This hypothesis implies that matriptase autoactivation should only be possible in cells that express both matriptase and prostasin, or if this is not the case, that there are multiple mechanisms for matriptase activation in different systems. We, therefore, set out to determine in matriptase autoactivation can be observed in five different matriptase-expressing human lines, which express no prostasin. The lines tested included three hematological cancer lines, Raji, Namalwa, and Ramos (Fig 3A) and two carcinoma lines, DU145 and Hep 3B (Fig 3B). PC3 human prostate cancer cells were also included in this test since they have been reported to express no prostasin [32] although we were able to detect very low levels of prostasin protein using our prostasin specific mAb (data not shown). Matriptase protein was clearly detected as a 70-kDa zymogen in lysates prepared from these cells by Western blot analysis (Fig 3, lanes 1, band *a*). When these cells were exposed to a pH 6.0 buffer for 20 min and lysates were prepared, a proportion of the matriptase was converted to complexes with HAI-2 in the hematological cancer cell lines (Fig 3A, lanes 2, band *b*) or with HAI-1 in the three carcinoma cell lines (Fig 3B, lanes 2, band *c*). These data suggest that matriptase can in fact undergo zymogen activation in the absence of, or in the presence of very low levels of prostasin, suggesting that prostasin is not required for and/or does not participate in the process of matriptase autoactivation, at least, in these cells.

### Prostasin activation occurs in the absence of matriptase and is not induced by exposure to an acidic environment

**Fig 4:**
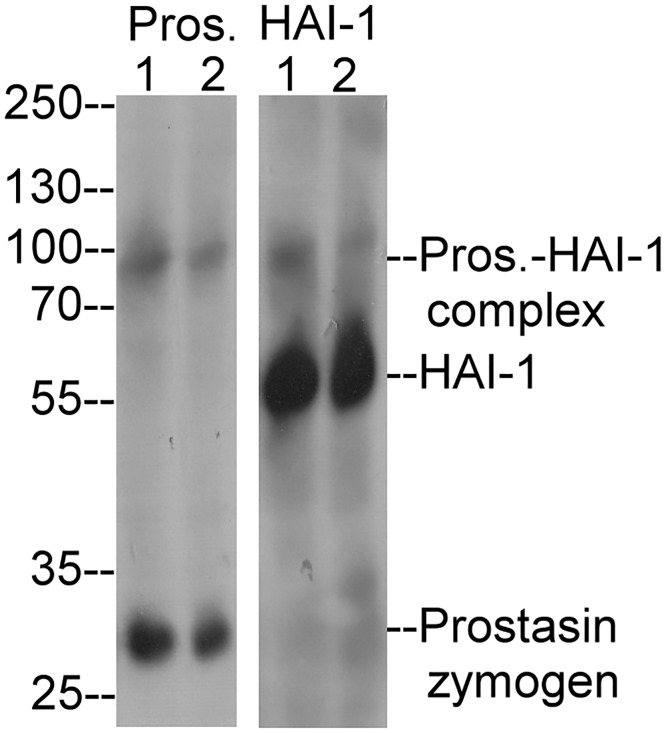
Prostasin does not undergo zymogen activation in response to exposure to a mildly acidic buffer in cells expressing no matriptase. ACHN human renal adenocarcinoma cells were treated with PBS (lanes 1) or pH 6.0 buffer for 20 min (lanes 2). Cell lysates were analyzed by Western blot analysis for prostasin species (*Pros*.) or for HAI-1 (*HAI-1*). The prostasin-HAI-1 complex (Prost.-HAI-1 complex), HAI-1 and prostasin zymogen are indicated. This experiment was conducted at least twice with the same profile observed. Representative data are shown.

In order to determine whether prostasin can undergo zymogen activation in the absence of matriptase, the human renal adenocarcinoma cell line ACHN was identified as a line that naturally expresses prostasin (i.e. not engineered to express prostasin) but no matriptase (Fig 4, Prost. lane 1). HAI-1 was also detected in the cells (Fig 4, HAI-1, lane 1). Low levels of prostasin zymogen activation appear to occur spontaneously in these cells as a small amount of prostasin-HAI-1 complex was detected by both prostasin and HAI-1 mAbs (Fig 4, lanes 1). This indicates that prostasin zymogen activation can occur in the absence of matriptase. Exposing ACHN cells to pH 6.0 buffer did not increase the levels of prostasin-HAI-1 complex (Fig 4, lanes 2), suggesting that prostasin does not exhibit the acid-induced autoactivation behavior exhibited by matriptase.

### Caco-2 cells express prostasin predominantly in the activated form and matriptase mainly in the zymogen form

**Fig 5:**
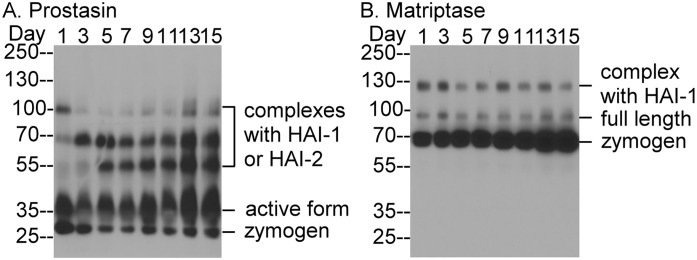
Human Caco-2 cells express prostasin predominantly in the activated form and matriptase primarily in the zymogen form. Human Caco-2 enterocytes were cultured and harvested at indicated dates post confluency. Cell lysates were analyzed by Western blot for prostasin species (*A*.) and matriptase species (*B*.). The composition of the various forms of prostasin and matriptase are indicated. The experiment was conducted twice with the same profile observed. Representative data are shown.

While examining the prostasin expression and zymogen activation status of a variety of human cell lines, we noticed that Caco-2 cells exhibited an unusual level of prostasin zymogen activation and an atypical inhibition pattern. The vast majority of the prostasin in these cells appeared to be in the activated form, and this was detected both as free active enzyme and in complexes with HAI-1 or HAI-2 (Fig 5A). A detailed characterization of these prostasin species can be found in our recent study (Shiao et al., manuscript submitted). Caco-2 cells that are allowed to become confluent in a culture vessel will over the next couple of weeks start to develop a more differentiated phenotype, developing tight junctions and forming a polarized cell monolayer. We were interested to find that prostasin zymogen activation status was maintained at very high levels throughout this process (Fig 5A). There seems to be a trend that the ratio of activated prostasin relative to total prostasin increases over the process of differentiation from around two thirds at the beginning to around nine tenths a few days after (Fig 5A). Matriptase was detected primarily in its zymogen form of 70-kDa (Fig 5B). The activated matriptase was detected in 120-kDa complex with HAI-1 at much lower levels. The ratio of activated matriptase relative to total matriptase appears to decrease over the process of differentiation (Fig 5B). Because it is prostasin zymogen that has been proposed to participate in the formation of the proposed reciprocal zymogen activation complex, it seems very unlikely that formation of the reciprocal zymogen activation complex is the mechanism for the activation of matriptase zymogen in Caco-2 cells, which maintain prostasin zymogen at very low levels and matriptase zymogen at high levels.

### Matriptase activity is not responsible for the significant constitutive prostasin zymogen activation exhibited by Caco-2 cells

**Fig 6:**
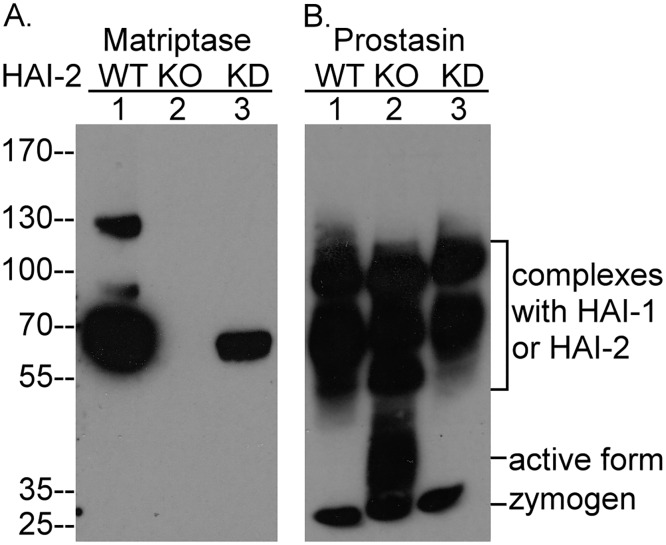
Caco-2 cells maintain high levels of prostasin zymogen activation regardless of matriptase expression. Parental Caco-2 cells (WT, lanes 1) and Caco-2 variants with reduced (KD, lanes 3) or ablated (KO, lanes 3) HAI-2 expression were analyzed by Western blot for matriptase species (*A*.) and prostasin species (*B*.). The different forms of prostasin are indicated. The immunblot analysis was conducted at least twice with the same profile observed. Representative data are shown.

As described above, prostasin zymogen activation appears to occur even if the cells do not express matriptase suggesting that, at least in those cells, matriptase is not required. The dispensable role of matriptase in prostasin zymogen activation was further emphasized by the observation that the levels of prostasin zymogen activation are not affected by alterations in the levels of matriptase protein expression in Caco-2 cells (Fig 6). We recently made the serendipitous observation in Caco-2 variants with reduced or ablated HAI-2 expression that we generated using CRISPR, the expression of matriptase protein was unexpectedly altered in parallel with the reduction in HAI-2 expression (Fig 6A). The mechanism by which HAI-2 expression alters matriptase expression is not currently known, but it is clear that the alterations in matriptase or HAI-2 expression had no effect on the extent of prostasin zymogen activation (Fig 6B). That prostasin zymogen activation is maintained regardless of the levels of matriptase protein in the system suggests that matriptase is not required or even involved in prostasin zymogen activation.

## Discussion

It has been suggested that prostasin is an activator of, or co-factor for matriptase zymogen activation. The former requiring prostasin enzymatic activity, the latter involving the formation of a reciprocal zymogen activation complex, for which prostasin proteolytic activity is dispensable. By examining protein expression and zymogen activation in response to mild acidification in a variety of human cells, we came to the conclusion that prostasin is not a matriptase zymogen activation co-factor at least, in those cells that do not express detectable prostasin. Presumably, cells that lack prostasin cannot form a reciprocal zymogen activation complex, and if matriptase still undergoes autoactivation in response to transient mild acidification in these cells, it does not involve this mechanism. A similar argument can be made regarding the possible role of prostasin enzyme activity in matriptase activation in cells that do not express measurable prostasin. It is possible that low levels of prostasin are present in the cells that we have examined and characterized as prostasin negative, but that the concentration present in the cells is below the level of detection of our antibodies. It seems unlikely, however, that such a low level of prostasin could be responsible for the extent and rapidity of acid-induced matriptase activity that we observe in these cells, particularly since prostasin activation does not seem to be acid-responsive. Furthermore, the lack of any relationship between the level of prostasin expression, or the degree of prostasin activation and matriptase zymogen activation is hard to explain if prostasin was so critically involved in matriptase activation, whatever the mechanism. If formation of a reciprocal zymogen activation complex were the mechanism for matriptase autoactivation in Caco-2 cells, one would expect a similar level of zymogen activation for matriptase and prostasin, whereas in fact the vast majority of prostasin has been activated but majority of matriptase remains in the zymogen form. Furthermore, since the majority of prostasin is already in the activated form, the level of prostasin zymogen is very low in Caco-2 cells. So low that it seems unlikely that it would be sufficient to form the reciprocal zymogen activation complex for matriptase autoactivation when Caco-2 cells are induced to activate matriptase by a pH 6.0 exposure. In fact, prostasin was detected at very low levels in many matriptase-expressing carcinoma cells, in which matriptase autoactivation can also be induced by acidification. One example is PC3 prostate cancer cells, which have been reported to not express prostasin. Low level of prostasin protein can be detected by our prostasin-specific mAb, and more importantly PC3 can be induced to activate matriptase by acidification.

The data generated with the Caco-2 cells is also hard to reconcile with a model in which it is the prostasin proteolytic activity that is directly responsible for matriptase activation. Caco-2 cells constitutively activate prostasin and retain significant levels of enzymatically active prostasin on the cell. This cell-associated active prostasin fails, however, to produce much matriptase zymogen activation. It is possible that differential subcellular localization of the proteins might be the reason for the lack of similar levels of matriptase zymogen activation to the levels of activated prostasin in Caco-2 cells. In our recent study, matriptase was detected primarily at the lateral membrane and prostasin primarily near apical brush border of the enterocytes of human intestine. The lack of a functional link at the zymogen activation levels due to this different subcellular localizations is at odds with the conclusion of a previous study, in which recombinant active prostasin was added to the cells to cause matriptase activation [13]. The mechanism of matriptase activation by active prostasin was initially suggested based on the relatively low level of matriptase activation that is induced when 50 nM of recombinant active prostasin was added to HaCaT human keratinocytes [13]. In order to convert matriptase zymogen to an active enzyme, cleavage at 614Arg-615Val is required [5,6,33]. Matriptase autoactivation via the interaction of matriptase zymogen molecules and intrinsic zymogen activation has also been proposed as the mechanism that executes this activational cleavage [4,8]. The autoactivation hypothesis does not exclude the possibility that matriptase zymogen activation can be mediated by other active proteases with trypsin-like activity regardless of the physiological relevance of this possibility. Trypsin has been shown to activate matriptase [34]. It is also not surprising to see the induction of matriptase activation when cells are incubated with exogenous active trypsin-like, serine proteases. In addition to prostasin, the addition of the recombinant active serine protease domains of hepsin or matriptase to HaCaT cells would also likely result in similarly low levels of matriptase zymogen activation [13]. Buzza et al., showed that very potent matriptase zymogen activation was induced when 5 nM recombinant prostasin was added to Caco-2 human enterocytes [12]. In this study matriptase zymogen activation was examined at 1, 3, and 5 hours post addition of recombinant prostasin to the cells. Interestingly and paradoxically, the levels and the kinetics of matriptase zymogen activation determined by the appearance of the matriptase serine protease domain appeared to increase over time from very low levels at 1 hr to very high levels at 3 and 5 hours, which mirrored the level of the disappearing 70-kDa matriptase zymogen over the course of the three time points. The induced matriptase activation kinetics also matched perfectly with the kinetics of the prostasin-induced development of transepithelial electrical resistance (TEER) in Caco-2 cells. Prostasin-induced TEER was increasing between hours 1–3 hour and stayed at a plateau between hours 4 and 5. Buzza et al., also showed that endogenous matriptase was required for the prostasin-induced development of TEER, leading them to conclude that prostasin stimulates enterocyte barrier formation via the activation of matriptase. These observations are, however, rather confusing since Caco-2 cells express very high levels of HAI-1 targeted to the basolateral plasma membrane [35]. Thus, the 5 nM prostasin added to the basal side of the cells should have encountered and been rapidly inhibited by HAI-1, which has been shown to be an important and very potent prostasin inhibitor [10,36] that can rapidly inhibit prostasin after its activation [9]. The rapid HAI-1-mediated inhibition of the exogenous active prostasin added to the cells in this study is entirely consistent with the very low levels of matriptase zymogen activation induced 1 hr post addition of active prostasin to Caco-2 cells in the study by Buzza et al., [12] and is also consistent with the low levels of matriptase activation in the similar study in HaCaT cells that first suggested a role for prostasin in matriptase activation [13]. Considering the much lower amount of recombinant prostasin used by Buzza et al., (5 nM versus 50 nM) and the rapid inhibition of prostasin by HAI-1, the very high levels of matriptase zymogen activation observed in the Buzza et al., study at 3–5 hr is not likely to be mediated by the active prostasin added to the cells, which should have been inhibited by HAI-1 on the basolateral plasma membrane of Caco-2 cells. Furthermore, a significant amount of the total cellular matriptase present in Caco-2 cells likely resides in intracellular pools, as is the situation seen in breast cancer cells [37]. Thus, since the exogenously added active prostasin should not have access to intracellular matriptase, any activation induced by the prostasin, should only deplete a portion of the cell-associated matriptase. The almost complete depletion of cell-associated matriptase zymogen observed at the later time points in the Buzza et al., study is, therefore, must be the result of some processes that caused matriptase zymogen activation both on the cell surface and inside the cells. Matriptase undergoes very dynamic metabolism following the induction of zymogen activation. In addition to the rapid inhibition of nascent active matriptase, both matriptase zymogen and the activated matriptase in complexes with protease inhibitors are rapidly shed from the cell surface into the extracellular milieu [15,18,20]. A significant decrease in the cellular levels of matriptase zymogen results not only from the conversion of the zymogen into activated matriptase, but also from shedding to the extracellular milieu. It seems possible to see the almost complete disappearance of cellular matriptase zymogen from Caco-2 cells 3–5 hr post prostasin treatment, but only if robust matriptase zymogen activation followed by shedding is induced.

The hypothesis that prostasin might be an important physiological substrate of matriptase was initially suggested by the great similarity between the epidermal defects observed in matriptase and prostasin knockout mice [1,2] and the lack of prostasin zymogen activation in the skin of matriptase knockout mice [3]. This apparent functional linkage observed in mouse skin does not, however, seem to apply in human skin due to the differential expression of matriptase and prostasin that is observed in the different layers of human skin, and during the process of epidermal differentiation. In human epidermis matriptase is expressed by the basal and spinous keratinocytes, whereas the granular keratinocytes express matriptase at negligible levels [38,39]. In contrast, prostasin is expressed primarily by the granular keratinocytes and not detected in the basal keratinocytes [40]. Nevertheless, matriptase and prostasin are co-expressed in several human epithelial cells in which induction of robust matriptase autoactivation does result in prostasin zymogen activation. The release of free active matriptase from the lipid bilayer biomembrane may provide active matriptase with access to and the ability to activate prostasin zymogen at the cell surface and/or in the secretory pathway. While this study suggests that prostasin may be a matriptase substrate, the physiological relevance of this interaction remains unclear. First of all, the huge amount of matriptase zymogen activation that can be induced by pH 6.0 buffer exposure is only seen in the absence of chloride [21]. In the presence of physiological concentration of sodium chloride, acid-induced matriptase zymogen activation is significantly attenuated [21]. Secondly, when matriptase zymogen activation is induced at more modest levels, such as in human mammary epithelial cells treated with sphingosine 1-phosphate, concomitant prostasin activation is negligible (Fig 2).

Our current study clearly indicates that the formation of a reciprocal zymogen activation complex is not necessary the mechanism for the zymogen activation of matriptase and prostasin, at least in the cell models examined here. The conclusion does not, however, suggest that there is no functional link between these two membrane serine proteases. Matriptase and prostasin share an inhibitory mechanism through the action of HAI-1 and HAI-2 [10]. Alteration in either of the two serine proteases could indirectly impact the other via HAI-1 and HAI-2. For example, prostasin has been reported to be involved in matriptase zymogen activation and shedding, and both processes were suggested to be HAI-2-associated in intestine [41]. This study of the functional link between matriptase and prostasin began with the observation that matriptase protein expression is reduced in HAI-2-deficient intestine in prostasin hypomorphic mice. The impact of HAI-2 deficiency on matriptase expression in mouse intestine was also mirrored in Caco-2 human enterocytes, in which reduced HAI-2 expression mediated using HAI-2 specific siRNAs caused a reduction in the levels of matriptase zymogen in both cell lysates and conditioned medium. The levels of prostasin protein appear to be not affected in this context, which is consistent with our observation in Fig 6, in which the levels of matriptase protein were reduced in proportion with the degree of HAI-2 reduction in Caco-2 cells. The Friis et al. study [41] further suggested that the reduced HAI-2 expression in Caco-2 cells resulted in enhanced matriptase activation and cell surface shedding, however, we do not agree that the data presented in their study convincingly support this conclusion. The level of matriptase zymogen activation was determined by assessing the level of matriptase serine protease domain (MTP-SPD) under reducing and boiled conditions, however, the level of MTP-SPD in HAI-2-reduced Caco-2 cell lysate appears to be the same as that in the control cells. The level of matriptase zymogen activation was also determined by assessing the level of matriptase-HAI-1 complexes, the size of which is 120-kDa in cell lysates, and 110-and 95-kDa in conditioned medium. Based on the levels of the 120-kDa matriptase-HAI-1 complex in the data presented, the control cells appear to exhibit higher levels of matriptase zymogen activation than the HAI-2-reduced cells. This would lead to the conclusion that matriptase zymogen activation is reduced by the reduction of HAI-2, which is contradictory to the conclusion reached in the paper. Furthermore, because the 25-kDa MTP-SPD is released from the 120-kDa matriptase-HAI-1 complex after the sample is reduced and boiled one would expect that the levels of the 25-kDa- MTP-SPD would correlate with the levels of the 120-kDa matriptase-HAI-1 complex. The difference between the cellular levels of MTP-SPD and cellular levels of 120-kDa matriptase-HAI-1 complex in the data presented is not discussed in this study. In the conditioned medium, the levels of MTP-SPD and the levels of matriptase-HAI-1 complexes are higher in the HAI-2-reduced cells than the control cells. Thus, Friis et al., apparently drew their conclusion based on the data generated using the conditioned medium but do not address the contradictory data from the cell lysates. Friis et al. also base their conclusion that HAI-2 reduction enhanced matriptase cell surface shedding on the higher levels of soluble activated matriptase species present in the conditioned medium from HAI-2-reduced Caco-2 cells versus the control cells. This led them to conclude that the HAI-2 reduction-enhanced matriptase shedding is responsible for the reduced cellular matriptase levels in the HAI-2 reduced cells. This conclusion is, however, not supported by the data presented that show that a higher level of matriptase zymogen is detected in the conditioned medium of the control cells than the HAI-2-reduced cells. Rather, these data would suggest that HAI-2 reduction suppresses matriptase shedding, at least, for the zymogen form. Thus, while our current study is consistent with the observations made by Friis et al regarding the impact of HAI-2 on matriptase protein levels, we believe that the underlying mechanism responsible for the reduction in matriptase levels requires further study.

It is worth noting that matriptase can spontaneously undergo zymogen activation in prostasin-negative cells as well as prostasin-positive cells, particularly when the cells are cultured at relatively high density or when differentiation is induced. The methods used in the current study, including exposing the cells to mildly acidic acid buffer, or treatment with lysophospholipid serve to accelerate this autoactivation process. Matriptase can undergo autoactivation when the cells are exposed to a pH 7.4 environment. The zymogen activation can be significantly accelerated by pH 6.0 [21]. Acidification is a common mechanism for the regulation of the zymogen activation of proprotein convertases when the proteases are trafficking through the secretory pathway, in which pH falls to below pH 6.0 in the secretory vesicles [42]. Mild acidification has also been shown to occur in many pathophysiological processes. The pH of urine in the distal tubules of the kidney can fall to below 5.5 as a result of the secretion of hydrogen ions and the reabsorption of bicarbonate [43]. The maintenance of an acidic pH plays essential roles in skin functions, particularly in stratum corneum integrity and cohesion, epidermal permeability barrier and antimicrobial barrier functions [44–46]. Sperm maturation and storage in the epididymis is also dependent on a mildly acidic environment [47]. In pathological contexts, tumor microenvironments are more acidic than normal tissues as a result of hypoxia, increased glucose metabolism, and the ineffective removal of acidic metabolites. Inflammation can induce interstitial acidification [48,49], and severe tissue ischemia causes extracellular acidosis [50].

In summary, defining the functional relation between matriptase and prostasin is challenging. The tight functional linkage in mouse skin, the shared inhibition mechanism by HAIs, the presence in human milk of the activated forms of both enzymes, and the simultaneous zymogen activation induced in cultured cells make it very likely that matriptase and prostasin do function as tightly coupled partners in some contexts. Our current study, however, indicates that prostasin is not needed for matriptase autoactivation and that active prostasin does not appear to be an important matriptase activator. While matriptase can activate prostasin, prostasin more typically undergoes zymogen activation without matriptase involvement through an as yet identified mechanism or protease. While matriptase and prostasin may still be functionally linked through some other mechanisms, their role in the zymogen activation of one another is at the least quite limited and context dependent, at least, in human cells. The discrepancy in the data presented here and the conclusions drawn by previous studies could result from the genuine physiological difference in the regulation of matriptase in human versus mouse skin and from the autoactivation of matriptase, which can be modulated by many factors, including the cellular chemical environment [21].
